# Isolation and identification of *Pandoraea *spp. From bronchoalveolar lavage of cystic fibrosis patients in Iran

**DOI:** 10.1186/s13052-019-0687-x

**Published:** 2019-09-02

**Authors:** Mohammad Tabatabaei, Mahdi Dastbarsar, Mohammad Ashkan Moslehi

**Affiliations:** 1 Faculty of Veterinary Medicine, Shiraz University, Shiraz, Iran; 2 Shiraz University of Medical Sciences, Shiraz, Iran

**Keywords:** *Pandoraea* spp., Cystic fibrosis, Bronchoalveolar lavage, PCR, Iran

## Abstract

**Background:**

*Pandoraea* species are gram negative, motile, non-spore forming, rod shaped and oxidase positive, obligate aerobes bacteria, and have one polar flagellum. Most of *Pandoraea* species are associated with lung infections in cystic fibrosis patients. Cystic fibrosis is the most prevalent autosomal recessive hereditary disease in the world that affects various organs of the body. The main important cause of death in these patients is lung involvement. This study was conducted to isolate and identify *Pandoraea* bacterium from bronchoalveolar lavage and sputum samples of cystic fibrosis patients in Shiraz, Iran.

**Methods:**

In this research 31 samples of bronchoalveolar lavage and sputum were examined by culture and PCR method. Then confirmed isolates were evaluated for susceptibility to different antibiotics and ability to produce biofilm.

**Results:**

The results of this study after cultivation, purification and DNA extraction led to the isolation of 4 *Pandoraea *bacterium by PCR using specific primers. Antibiotic susceptibility test were indicated all isolates were resistant to gentamicin, amikacin and imipenem and susceptible to ciprofloxacin, trimethoprim-sulfumethoxazole, piperacillin and tetracycline. Ability to create biofilm was indicated by some of *Pandoraea* isolates. According to findings of this study, ability to synthesis biofilm by *Pandoraea* isolates and resistance to some antibiotics are very important.

**Conclusions:**

Our study notes the role of *P*. *pnomenusa *as an emerging pathogen that can cause chronic lung colonization in CF patients. Identification tools need to be accurate and must be based on molecular techniques. Also our findings should raise awareness about antibiotic resistance in cystic fibrosis patients in Iran and ability of including bacterial agents to produce biofilm is an alarm for public health. Thus clinicians should exercise caution about finding of clinical relevance of this pathogen to the infection and prescribing antibiotics, especially in cases of children infections.

## Introduction

The genus *Pandoraea* is composed of aerobic, non-sporeforming, non-nitrate reducing, non-lactose fermenting, and gram negative rods shaped bacteria, with polar flagella. They have been grown at 30–37 °C in 0.5 and 1.5% NaCl or on Drigalski agar, assimilate D-gluconate, L-malate, and phenylacetate, and have catalase, acid and alkaline phosphatase and leucine arylamidase activity [1]. Different species of *Pandoraea* have been described: *P. apista*, *P. norimbergensis*, *P. pnomenusa*, *P. pulmonicola*, *P. sputorum*, *P. fibrosis*, *P. terrae, P. oxalativorance, P. faecigallinarum, P. vervacti, P. thiooxidance* and four unnamed genomospecies. The majority of isolates have been isolated from respiratory samples from patients with cystic fibrosis (CF) or other underlying chronic lung disease but can also be isolated from other clinical samples (including blood) and from the environmental samples such as soil, food, sea, and drinking water [1–5]. Its closest phylogenetic relative is the genus *Burkholderia* and, like members of the genus *Burkholderia*, the *Pandoraea* spp. are emerging important respiratory pathogens, particularly in people with cystic fibrosis (CF). Species in this genus are often misidentified as *Burkholderia cepacia* complex (Bcc) or *Ralstonia* species owing to overlapping biochemical profiles without differences that reliably distinguish between species [6]. It is because of these limitations that reliable identification requires 16S ribosomal DNA sequence analysis.

Cystic fibrosis is caused by mutations in the CFTR (cystic fibrosis transmembrane conductance regulator) gene [7]. The commonest mutation is the deletion of phenylalanine at codon 508 (phe508del). This occurs in about 70% of patients with cystic fibrosis. The primary function of the CFTR protein is as an ion channel that regulates liquid volume on epithelial surfaces through chloride secretion and inhibition of sodium absorption. The commonly accepted explanation for airway disease in cystic fibrosis is the “low volume” hypothesis. A reduced volume of airway surface liquid causes failure of mucociliary clearance, the lungs’ innate defense mechanism [8]. The mucociliary dysfunction means that a patient with CF cannot effectively clear inhaled bacteria. In addition, there is an excessive inflammatory response to pathogens. For a given bacterial load, a person with CF will have up to 10 times more inflammation than a person with a lower respiratory tract infection but without the disease. The reasons for the excessive inflammatory response to pathogens are not fully understood. The abnormal composition and secretion of mucus may also be important. At birth, the airway is uninfected and probably uninflamed [9], but the end result of the abnormalities described above is irreversible airway damage with bronchiectasis and respiratory failure in most patients. Ion and water abnormalities may also cause disease in other epithelia-lined organs.

The main source of morbidity and mortality in CF patients, is the decline in the pulmonary function subsequent to pathogenic colonization with non-fermenting Gram negative bacteria (NFGNB) that they encounter throughout their lives.CF patients are particularly susceptible to infections caused by specific bacterial pathogens such as *Pseudomonas aeruginosa, Staphylococcus aureus* and *Haemophilus influenzae* [10].

Although *Pandoraea* species have also been isolated from sputum samples of CF patients, there is still very little known about their mechanisms of pathogenicity or their roles in CF lung disease [11]. In addition, *Pandoraea* isolates have been recovered from both CF and non-CF patients from a variety of clinical samples including blood, sputum, urine, the upper airways and lung tissue [12]. The recovery of *Pandoraea* isolates from the blood of patients indicates that this organism is capable of invading tissue [13, 14]. Antibiotic therapy for treatment of infection is difficult due to the limited number of antibiotics to which these species are susceptible: tetracycline, imipenem and trimethoprim–sulfamethoxazole [15].

However, the clinical significance of colonization with these organisms remains unclear [13] and there are limited and conflicting data available on the clinical outcome of patients colonized with *Pandoraea*.

Whether *Pandoraea* spp. is truly pathogenic is not yet fully understood, there is evidence that infection with *Pandoraea* spp. invokes a host inflammatory response. A number of virulence factors have been associated with clinical virulence of *Pandoraea* spp. and Bcc, including stimulation of pro-inflammatory cytokine secretion known to cause lung tissue damage (IL-6 and IL-8), biofilm formation and, in the case of Bcc, the ability to invade lung epithelial cells, which may contribute to the persistence of the strains in the CF lung [16–20]. Indeed, biofilm formation by *Pandoraea* spp. and Bcc has been specifically associated with increased resistance to antibiotics and maintenance of the bacteria in the lung [21–24]. Here, we report the results of isolation and identification of *Pandoraea* spp. and their ability to form biofilms in vitro, in order to gain insight into their virulence potential in CF lung infections.

In the clinical microbiology laboratory, identification to the species level and differentiation of *Pandoraea* species from organisms belonging to the Bcc, *R. pickettii*, or *R. paucula* may be problematic [1, 6, 25]. To aid in the identification of these organisms, we used PCR-based identification strategies based on the *16S rRNA* gene (rDNA).

## Materials and method

### Sample collection

From November 2017 to August 2018, following the sedation of CF patients in Namazi hospital (Shiraz, Iran), 31 samples of bronchoalveolar lavage and sputum were collected. Then the samples were transferred to the bacteriology laboratory, faculty of veterinary medicine of Shiraz University, in cold condition. The study was approved by the Ethics Committee of Shiraz University of Medical Sciences.

### Culture and isolation

To obtain strains and to compare culture and PCR results, cultures were performed initially as enrichment cultures. Upon arrival, bronchoalveolar lavage and sputum were cultured in BHI broth and incubated aerobically at 37 °C for 24 h. Initial enriched cultures were transferred to the enrichment culture plates by serial streaking the cultures on sheep blood agar and incubated aerobically at 37 °C for 24–48 h.

Then, a loop full of the identified colonies were streaked on a new blood agar and incubated aerobically at 37 °C for 24 h. Next, from culture positive plates, typical colonies were subjected to gram’s staining to study staining reactions and cellular morphology under light microscope. Mixed and gram-negative bacteria were further sub cultured with due care, on both blood agar and MacConkey agar plates for final identification. The growth of typical colonies on both blood agar and MacConkey agar was characterized using blood agar for the presence and type of haemolysis, and the general appearance of colonies (morphology, color, shape, size and consistency) and the MacConkey agar for the ability to ferment lactose. Pure cultures of single colony type were further analyzed by catalase and oxidase tests. Confirmation of bacteria to species level was aided by using the biochemical tests including, metabolism of sugars such as glucose, fructose, lactose and tests for metabolic end products such as nitrate reduction, unease and citrate activity, growth on cetrimide agarat 42 °C and O/F medium following standard procedures. For final confirmation, isolates evaluated by two PCR reactions. A first PCR assay was designed to identify all *Pandoraea* spp. Subsequent PCR assays were designed to identify individual *Pandoraea* species.

### DNA extraction

A few colonies from the phenotypically characterized pure cultures of *Pandoraea* from 24 to 48 h growth on blood agar plates were transferred into 1.5 ml Eppendorf tubes. Total DNA was prepared using the Gram negative DNA extraction kit (Cinagen, Iran). The protocol for Gram negative bacteria as described in the kit was followed for extractions. The extracted DNA were determined to be of good quality and DNA concentration was measured using Nanodrop (10,000 V 3.52). DNA concentrations were adjusted to 15 ng μL^− 1^ before PCR amplification. Finally, extracted DNA stored at −20 °C for further use.

### Master mixture of PCR

The oligonucleotide primers used in this study were synthesized by Cinagen Company (Iran). The forward and the reverse primers sequences are as PanF (5´GGGCTYAACCTGGGAACTGCATTC3´), and PanR (5´CGRYTTGGCRRCCCTCTGTACCG3´) [26]. Also PCR evaluations have been done with two universal primers for amplification of *16S rRNA* gene. The reaction mixture solution for PCR was prepared using 2 μl of 10x PCR buffer, 1.5 μl (1.5 mM) MgCl_2_, 1.2 μl (200 mM) dNTPs, 1.2 μl (100 nmol) each primer of *Pandoraea*, 0.2 U of *Taq* DNA polymerase, 14.7 μl H_2_O, and 3 μl of DNA template to have a final volume of 25 μl. Using 0.2 ml thin wall microtubes. After initial denaturation (at 94 °C for 5 min), amplification conditions were, denaturation at 94 °C for 1 min, annealing at 60 °C for 45 s, and extension at 72 °C for 1 min. This was repeated for 30 cycles in a Block assembly 96G thermocycler with a hot top assembly (Analytic Jena, Germany), with a final extension of 72 °C for 10 min and the PCR products remained in the thermal cycler at 4 °C until they were collected. All DNA isolation procedures were carried out in a Class II biological safety cabinet in a room geographically separate from that used to set up reaction mixes and also from the ‘post PCR’ room, in order to prevent crossover contamination by extraneous nucleic acids and in accordance with good molecular diagnostic practice. All reaction mixes were set up in a PCR hood in a room separate from that used to extract DNA, and from the amplification and post-PCR room, in order to minimize contamination. A negative control consisting of all component of reaction mixture except the DNA template was included in all PCR. Positive controls were included in the PCR from colonies that were confirmed according to NCBI gene bank sequencing. In order to evaluate the PCR results electrophoresis in agarose gel was carried out. PCR amplicons were assessed by loading 8 μl of the PCR product plus 2 μl of loading buffer into separate wells of a 1% (w/v) agarose gel (Agarose I; Cinnagen, Iran) containing safe stain. A molecular weight marker (50 bp; Cinnagen, Iran) was loaded into the first well to determine the size of the amplified fragments. The gel was immersed in TBE buffer and subjected to a voltage difference of 100 V that led to separation of the fragments. Visualization was undertaken using an ultraviolet transilluminator (BTS-20, Japan), and the resulting image was captured by a computer software program (Alpha Ease; Alpha Innotech).

For the final confirmation of isolates of *Pandoraea,* PCR products of Pan primers for some samples according to the Macrogene Company’s instructions were prepared at 50 μl volume and were transferred for sequencing. In addition, *16S rRNA* gene sequencing for species identification was applied for some of the *Pandoraea* isolates. The sequences were compared with previously published *16SrRNA* (GenBank accession no.NR-152002), *Pandoraea* gene sequences.

### Antimicrobial susceptibility testing

Antimicrobial susceptibility testing for *Pandoraea* spp. isolates was performed by disk diffusion method according to the Clinical and Laboratory Standards Institute (CLSI) [27]. Disk diffusion method was performed on Muller-Hinton agar (Merck, Germany), using an inoculum of 10^5 ^CFU/ml. Antibiotic disks (Padtan teb, Iran) including amikacin, ciprofloxacin, trimethoprim- sulfumethoxazole, gentamicin, piperacillin tetracycline and imipenem. These antibiotic disks were then placed on agar plates and incubated at 37 °C for 24 h. *Escherichia coli* (ATCC 25922) and *Pseudomonas aeruginosa* (ATCC 27853) were used as quality controls strain in each susceptibility determination. The diameter of inhibition zone was measured in millimeters and isolates were scored as susceptible or resistant by comparing results with values recommended on standard charts.

### Biofilm assay

In order to evaluate the ability to create biofilms in *Pandoraea* spp. isolates used the method of Merritt et al. and Wakimoto model [28, 29]. Briefly, 100 μl of each diluted culture was relocate into individual wells of microtiter dish and grown at 37 °C overnight. All wells were stained with 125 μl of 0.2% (w/v) crystal violet solution for 10 min at room temperature. The excess stain was washed twice with distilled water and the plate was allowed to air dried. 200 μl of 95% ethanol was added to solubilize the stained biofilm and incubated for 10–15 min at room temperature. 125 μl of each well was transferred into a new microtiter dish. Finally, the optical density (OD) of each sample was measured at 570 nm by a spectrophotometer. The isolates with the optical density higher than 0.2 were considered as biofilm former isolates.

## Results

Identification of the bacterial species was made by observation of their colonial morphology, gram staining reaction and biochemical characteristics according to Coenyeet al. (2000). Through culture 8isolates of *Pandoraea* with small gray circle colonies without haemolysis was observed that were rod shapes in gram staining. Catalase and oxidase tests were positive [1].

In this study, *Pandoraea* strains were isolated from clinical specimens, as in previous studies including bronchoalveolar lavage and sputum of CF patients. Also, most isolates were involved in polymicrobial infections, including *Staphylococcus, Pseudomonas, Neisseria flavorans* (according to DNA sequencing), *Delftia acidovorans* (according to DNA sequencing) and *Klebsiella.*

**Fig. 1:**
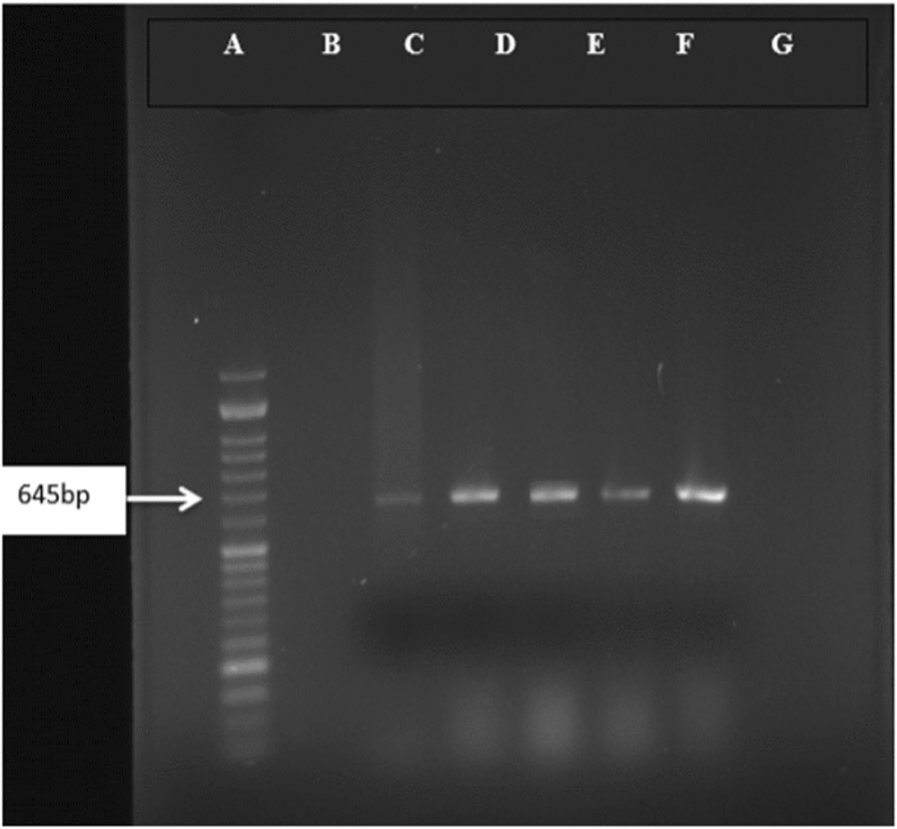
PCR amplification profile of *Pandoraea* spp*.* from DNA isolated directly from samples with PAN primers. Lane **a**: 50 bp DNA marker, Lane **b**: Negative control, Lane C: Positive control and Lanes D-G: Positive samples (645 bp amplicon size). It should be noted that 4 samples were absolutely homologues with documented genomic sequencing of *Pandoraea* in NCBI genomic bank

**Fig. 2:**
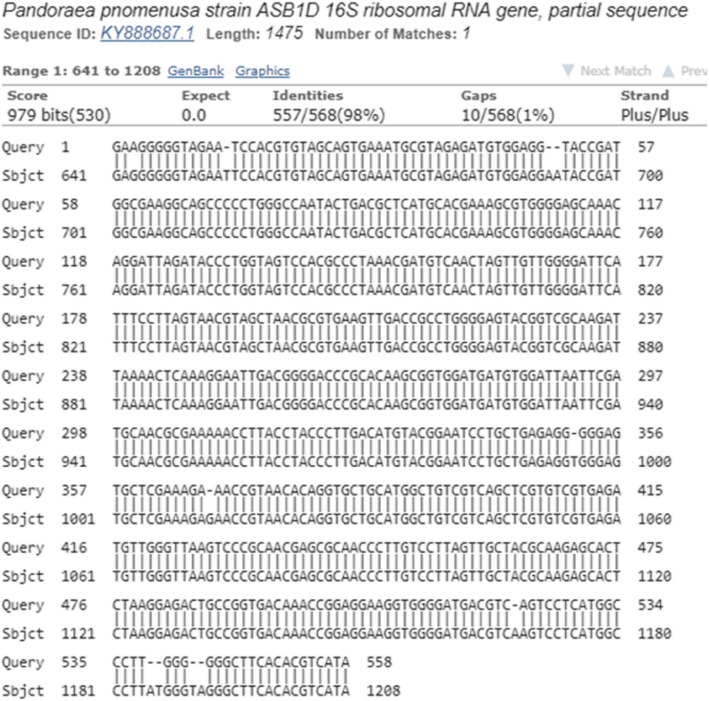
Genomic sequencing of *Pandoraea* documented in NCBI genomic bank

When these culture positive isolates evaluated by PCR, using the stated primers, all the samples tested for the presence of *Pandoraea* were identified as 100% positive (Figs. 1, 2).

### Antimicrobial susceptibility

**Fig. 3:**
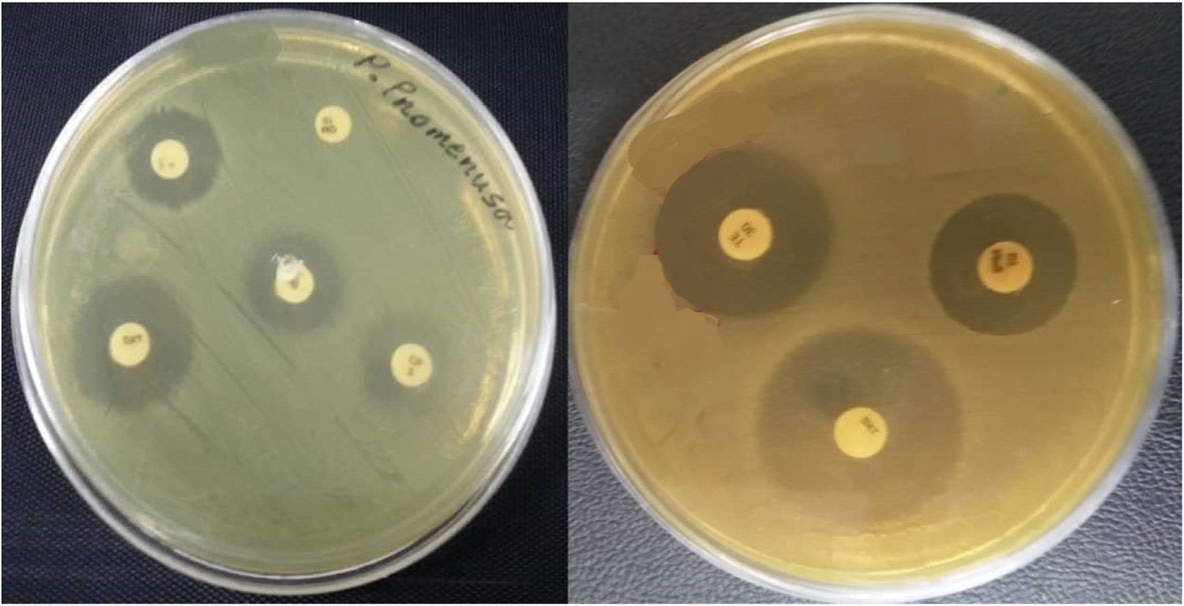
Antibiogram results of *Pandoraea* isolates

In this study susceptibility to antibiotics was detected using 7 usual antibiotics prescribed for treatment of chronic lung infection including amikacin, ciprofloxacin, trimethoprim-sulfumethoxazole, gentamicin, piperacillin, tetracyline and imipenem. Antibiotic susceptibility test were indicated all isolates were resistant to gentamicin, amikacin and imipenem and susceptible to ciprofloxacin, trimethoprim-sulfumethoxazole, piperacillin and tetracycline (Fig. 3).

### Biofilm assay

Our target in this study was phenotypical survey of biofilm synthesis ability. According to tests carried out on four *Pandoraea* isolates evaluated for the ability to create biofilms in LB, TSB and BHI medium, one isolate create strong biofilm in BHI medium, one create strong biofilm in TSB medium, but all four isolate create weak biofilm in LB medium. This results indicate that biofilm formation can differ according the culture media.

## Discussion

Cystic fibrosis (CF) is the most common life-limiting autosomal recessive disease among people of European heritage [30]. In the United States, about 30,000 individuals have CF; most are diagnosed by six months of age. In Canada, about 4000 people have CF [31]. Around 1 in 25 people of European descent, and one in 30 of Caucasian Americans [32], is a carrier of a CF mutation. Although CF is less common in these groups, roughly one in 46 Hispanics, one in 65 Africans, and one in 90 Asians carry at least one abnormal CFTR gene [33, 34]. Ireland has the world’s highest prevalence of CF, at one in 1353 [35].

CF is a common genetic disease. Due to this condition, mucosal secretions in the lung can cause recurrence and persistent respiratory infections. A number of opportunistic pathogens settle in these patients. In most cases, in chronic infections, *Pseudomonas*, *Burkholderia*, as well as *Pandoraea* spp. have been isolated from the lung of CF patients, which play role in emerging pathogenesis. *Pandoraea* spp. are considered emerging pathogens in the context of CF and are difficult to identify by conventional biochemical methods. Most *Pandoraea* spp. are present in the lung of CF patients, lung infections, and oral and dental infections. According to the studies conducted to data on patients with CF and lung infection diseases, opportunistic pathogens that have been involved in causing diseases including *Pseudomonas*, *Burkholderia* and *Pandoraea* species, have been isolated and identified [29, 36–38].

One group of bacteria currently considered to be emerging CF pathogens belongs to the genus *Pandoraea*. The genus *Pandoraea* was described by Coenye et al. (2000) to differentiate them from other already well-known CF pathogens, including *Pseudomonas* and two closely related Gram-negative rods, *Burkholderia* and *Ralstonia* species. In fact, phenotypic methods used by many microbiology laboratories commonly lead to the misidentification of *Pandoraea* species as either *Burkholderia* or *Ralstonia* species [2, 4].

*Pandoraea* infection led to the production of high levels of antibodies, and to a worsened CF lung disease [4, 10, 39]. After first colonization, *Pandoraea* spp. were able to chronically colonize the CF respiratory tract (CFRT) [10, 40–42], were transmissible between patients [4, 37] and can produce severe lung diseases and bacteremia [12, 43]. The pathogenicity appears mainly supported by a pro-inflammatory response induction significantly greater than with *P. aeruginosa* [19, 44] and the treatment may be complicated by multidrug resistance conferred by carbapenem hydrolyzing oxacillinases [19]. The potential involvement of *Pandoraea* in complex interactions between microorganisms within the CF airways was also suggested [44].

In current study from 31 samples of bronchoalveolar lavage and sputum, by culture method eight *Pandoraea* bacteria were isolated and identified. These bacteria were isolated and purified using conventional diagnostic methods such as culture and biochemical tests. But finally by PCR assay just 4 isolates confirmed as *Pandoraea*. Also, in addition to *Pandoraea* spp. other bacteria including *Staphylococcus, Pseudomonas, Neisseria flavorans*, *Delftia acidovorans *and *Klebsiella* were also isolated.

In 2001, Coenye et al. identified and investigated species of *Pandoraea* bacteria. In this study, from 123 samples, *Pandoraea* (69), *Burkholderia* (30), *Ralstonia* (9) and *Pseudomonas aeruginosa* (5) were isolated and identified. For the first time, PCR testing was performed to identify the member of genus *Pandoraea*, which were able to detect *P. apista*, *P. pnomenusa*, *P. sputorum, P. pulmonicola* and *P. norimbergensis* [26].

Daneshvar and colleagues in 2001 examined the cellular effects of *Pandoraea* spp. on lung cells in CF patients. The study also found that it was possible to isolate the *Pandoraea* from the cultures of patient’s blood who did not have cystic fibrosis [2].

In the study of Spreet et al., conducted in Canada in 2002, from a total of 447 CF patients in 8 provinces included 5 *Pandoraea* (1.1%), 412 *Burkholderia* (92.6%) and 5 *Ralstonia* (1.1%) bacteria, using culture techniques, biochemical tests and molecular techniques including PFGE, RAPD and RFLP were identified and isolated [45].

In 2003, Jorgensen et al., examined the epidemic of *P. apista* in patients with cystic fibrosis. According to the results of this study, it has been shown that *P. apista* should be added to the ever increasing list of pathogens that can cause chronic lung infections in CF patients [43].

In 2008, Karher et al. examined the pathogenic and genetic characteristics of *Pandoraea* species in lung epithelial cells. In this study, 17 *Pandoraea* bacteria were isolated including 5 species: *P. apista*, *P. pnomenusa*, *P. sputorum, P. pulmonicola* and *P. norimbergensis* [38].

In a study conducted by Panickar and David (2015) from October 2012 to September 2014 on outpatient, and lavage samples from 182 CF children treated at the Royal Manchester Hospital, 5(3%) were *Burkholderia,* 17(19%) *Exophiala*, 32(18) *Achromobacter* and 32 (18%) *Rhodotorula*, 18 (10%) *Achromobacter*, 1 *Ralstonia* and 6 (3%) *Pandoraea* were identified. This study showed that the number of *Pandoraea* and *Achromobacter* bacteria in CF children is increasing [39].

Antibiotic treatment of infections caused by *Pandoraea* species is difficult because it has been demonstrated resistant against a wide range of antibiotics such as ampicillin, cefazolin, piperacillin, azithroman, broad- spectrum cephalosporins and aminoglycosides, and show a different response to quinolones, trimethoprim-sulfumethoxazole, colistin and carbapenems. *Pandoraea* species are resistant to most antibiotics, including beta-lactams and aminoglycosides. The bacteria in the subjects evaluated moderate sensitivity to imipenem, doxycycline and ceftriaxone, and an unusual antibiotic susceptibility pattern to carbapenem was also found in *Pandoraea* bacteria and resistance to meropenem and sensitivity to imipenem has been reported.

The result shows that for antibiotic treatment of *Pandoraea* infection, antibiogram test for each person has been done individually. Then if a person is allergic to imipenem they can use carbapenem. All *Pandoraea* species except *P. apista*, G9278 are resistant to amikacin and cefazolin antibiotics, most of which are resistant to broad-spectrum cephalosporins, azithroman and piperacillin. Also, these organisms are resistant to aminoglycosides such as gentamicin, tobramycin and amikacin, but their susceptibility to fluroquinolones is different [40].

Puges et al. (2015), reported that *P. sputorum* was susceptible to imipenem and resistant to meropenem. This discrepancy has already been described by several authors and is because of meropenem-hydrolyzing β-lactamases. Some strains are resistant to both imipenem and meropenem, and an imipenem-hydrolyzing oxacillinase named OXA-62 has been identified in *P. pnomenusa* [45].

In this study susceptibility to antibiotics was detected using 5 antibiotics including amikacin, ciprofloxacin, trimethoprim-sulfumethoxazole, gentamicin and piperacillin. Antibiotic susceptibility test were indicated all isolates were resistant to gentamicin, imipenem and amikacin and susceptible to ciprofloxacin, trimethoprim-sulfumethoxazole, tetracycline and piperacillin.

Biofilms are microbial society encased in extracellular polymeric substances (EPS) [18]. Biofilm formation symbolizes a protective mode of growth that allows microorganisms to survive in hostile environments [46]. Biofilm is responsible for persistent in chronic infections, due to their inherent resistance to antimicrobial agents. Biofilms are shown as being resistant to killing by a broad range of antimicrobial agents [47]. Some bacteria such as *P. aeruginosa* secrete the exopolysaccharide alginate during infection of the respiratory tract of individuals afflicted with cystic fibrosis and chronic obstructive pulmonary disease [48]. Biofilm production has been considered to be an important determinant of pathogenicity in most infections. In this study all 3 evaluated isolates showed strong biofilm formation ability.

## Conclusions

In conclusionsthe results of our study indicate that *Pandoraea* species can be isolated from bronchoalveolar lavage and sputum cultures of CF patients. The correct identification of this bacterial species presents a challenge for diagnostic microbiology laboratories. Our study supports the use of genotypic methods to augment routine phenotypic evaluation. The combined use of the two PCR assays described will allow the identification of most *Pandoraea* species encountered in bronchoalveolar lavage and sputum cultures of CF patients. Most importantly, the use of these assays will substantially reduce the misidentification of *Pandoraea* spp. as Bcc. These tests will be a valuable adjunct in the evaluation of CF bronchoalveolar lavage and sputum culture isolates and will allow more precise study of the prevalence and natural history of human infection by this emerging pathogen. Finally our findings should raise awareness about antibiotic resistance in CF patients in Iran and their ability to produce biofilm is an alarm for public health. Thus clinicians should exercise caution in prescribing antibiotics, especially in cases of children infections.

## Data Availability

Data sharing not applicable to this article as no datasets were generated or analyzed during the current study.

## Abbreviations

ATCC: American type culture collection
Bcc: *Burkholderia cepacia* complex
BHI: Brain heart infusion
bp: base pair
CF: Cystic fibrosis
CFTR: Cystic fibrosis transmembrane conductance regulator
CFU/ml: Colony forming units/ milliliter
CLSI: Clinical and Laboratory Standards Institute
DNA: Deoxyribonucleic acid
dNTPs: Deoxyribonucleic triphosphates
EPS: extracellular polymeric substances
LB: Luria bertoni
ml: Milliliter
NCBI: National Center for Biotechnology Information
ng μL-1: nanogram/ microliter
nm: nanometer
O/F: Oxiation/ Fermentation
OD: optical density
PCR: Polymerase chain reaction
phe508del: Phenylalanine 508 Deletion
rDNA: ribosomal Deoxyribonucleic Acid
rRNA: ribosomal ribonucleic Acid
*Taq*: *Thermus aquiticus*
TBE: Tris Borate EDTA (Ethylenediaminetetraacetic acid)
TSB: Triptic soy broth
w/v: Weight/Volume
